# Inflammation as a therapeutic target for osteoarthritis: A literature review of clinical trials

**DOI:** 10.1007/s10067-024-07042-y

**Published:** 2024-07-03

**Authors:** Rui Zhu, Haonan Fang, Junjie Wang, Liru Ge, Xiaoyue Zhang, Dawn Aitken, Guoqi Cai

**Affiliations:** 1 Department of Epidemiology and Biostatistics, School of Public Health, Anhui Medical University, Hefei, 230032 Anhui China; 2 Menzies Institute for Medical Research, University of Tasmania, Hobart, TAS 7000 Australia

**Keywords:** Inflammation, Osteoarthritis, Phenotype, Synovitis

## Abstract

The burden of osteoarthritis (OA) is rapidly increasing with population aging, but there are still no approved disease-modifying drugs available. Accumulating evidence has shown that OA is a heterogeneous disease with multiple phenotypes, and it is unlikely to respond to one-size-fits-all treatments. Inflammation is recognized as an important phenotype of OA and is associated with worse pain and joint deterioration. Therefore, it is believed that anti-inflammatory treatments may be more effective for OA with an inflammatory phenotype. In this review, we summarized clinical trials that evaluated anti-inflammatory treatments for OA and discussed whether these treatments are more effective in inflammatory OA phenotypes compared to general OA patients.

**Supplementary Information:**

The online version contains supplementary material available at 10.1007/s10067-024-07042-y.

## Introduction

Osteoarthritis (OA) is the most common joint disease affecting approximately 240 million people worldwide [1]. The number continues to increase with population aging and rising obesity rates, making OA an unignorable public health problem. However, no approved treatments are available to prevent or even retard OA disease progression [2]. Current treatments focus on relieving symptoms but the efficacy and safety of analgesics and non-steroidal anti-inflammatory drugs (NSAIDs) are often not desirable [3], especially in the long-term [4].

Accumulating evidence has shown that OA is a heterogeneous disease with multiple phenotypes [5, 6], and defining OA phenotypes is commonly based on characteristics of joint tissues (e.g., bone, cartilage, synovial tissue) and clinical assessments (e.g., comorbidity, symptoms, biochemical tests). Previous studies have summarized potential OA phenotypes such as bone phenotype, metabolic syndrome phenotype, pain phenotype, and inflammatory phenotype [6, 7]. These phenotypes are largely different in many aspects, making one-size-fits-all treatments unlikely. Among these OA phenotypes, inflammatory OA is a common type (prevalence approximately 16% to 60% in different samples [7, 8]) and is usually characterized by overexpression of inflammatory cytokines, and the increased signal intensity on ultrasound (US) or magnetic resonance imaging (MRI) that results from inflammation of the joint lining (synovitis) and joint fluid (effusion), termed “effusion-synovitis”. Epidemiological studies have shown that effusion-synovitis may be a source of OA symptoms and predict both symptomatic and structural progression of OA [9]. Therefore, it is believed that treatments targeting inflammation can delay or prevent cartilage damage and osteophyte formation. This suggests that inflammation is a therapeutic target for the inflammatory phenotype of OA and that anti-inflammatory treatments are more likely to achieve desirable outcomes. In this review, we summarized clinical trials that evaluated anti-inflammatory treatments for OA and discussed whether these treatments are more effective in inflammatory OA phenotypes compared to general OA patients.

## Literature research strategy

**Fig. 1:**
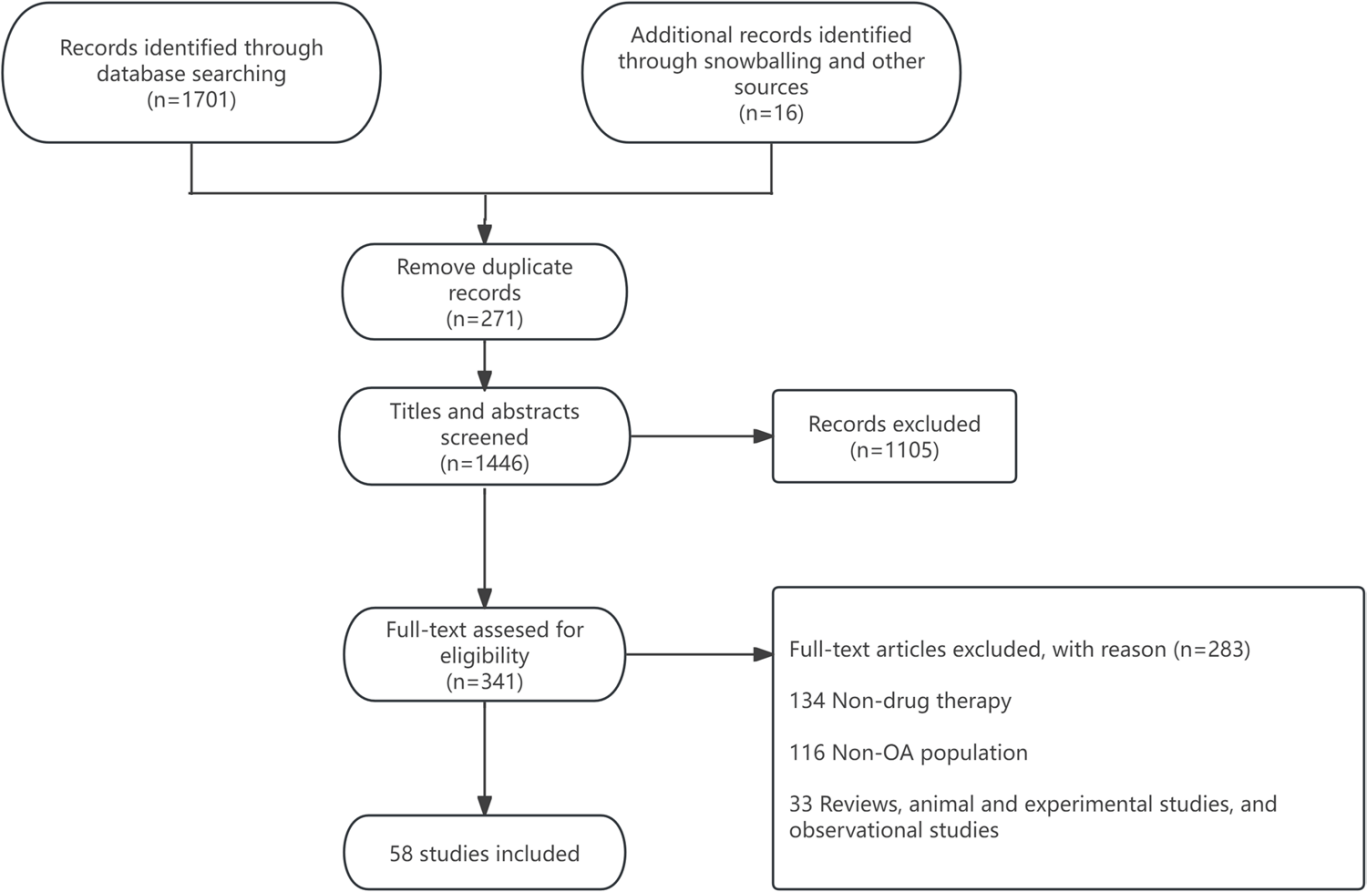
Study flow chart

We adopted a hybrid search strategy that included both database searches and snowballing. We searched PubMed and Web of Science from inception to December 2023 using search terms including “OA”, “osteoarthritis”, “inflammation”, “synovitis”, “effusion”, “therapy”, and “treatment” for clinical trials. The bibliography of the included studies was also checked for additional studies. Two reviewers (RZ and HF) independently reviewed the identified articles and conducted risk of bias assessment according to the Cochrane Collaboration's tool for eligible trials [10], with disagreement discussed with a third author (GC). Clinical trials evaluating the effect of anti-inflammatory treatments for OA with or without an indicator of inflammation were included. We excluded reviews, animal and experimental studies, and observational studies. After study selection, 58 articles related to this review were included (Fig. 1, Tables 1, 2, and 3). Overall, 65% trials showed a low risk of bias (Supplementary Table 1).

**Table 1:** Disease-modifying antirheumatic drugs (DMARDS) for the treatment of osteoarthritis

Studies	Participants	Design	Interventions	Conclusion
MTX				
Pavelka[11], 2006	n = 21, erosive hand OA	Open-label (single arm, follow-up time unclear)	10 mg of MTX per week	MTX decreased pain and morning stiffness, but not functional indices, number of tender and swollen joints
Ferrero[12], 2021	n = 64, erosive hand OA	RCT (12 months)	10 mg of MTX per week or placebo	MTX showed no effect on pain relief at 3 or 12 months
Wang[13], 2023	n = 97, hand OA with synovitis	RCT (6 months)	20 mg of MTX per week or placebo	MTX reduced pain and stiffness but did not function over 6 months in hand OA with synovitis
Enteshari-Moghaddam[14], 2019	n = 100, moderate to severe knee OA	RCT (6 months)	7.5 mg of MTX increased to 15 mg weekly, or placebo	MTX reduced pain severity and improved functional status and quality of life
Holanda[15], 2007	n = 58, knee OA	RCT (4 months)	7.5 mg of MTX weekly or placebo	MTX did not ameliorate symptoms or reduce functional limitation but showed a tendency to reduce the consumption of analgesics
Kingsbury[16], 2019	n = 155, clinical and radiographic knee OA	RCT (12 months)	10 mg of MTX increased to 25 mg weekly, or placebo	MTX improved NRS knee pain (adjusted treatment difference -0.83 (95% CI -1.55 to -0.10), WOMAC stiffness and function, but not WOMAC pain at 6 months compared to placebo. Treatment benefits were reduced by 12 months
Wenham[17], 2013	n = 30, knee OA with effusion synovitis	Open-label (single arm, 24 weeks)	7.5 mg of MTX increased to 20 mg per week	43% achieved ≥ 30% reduction in pain VAS, 23% achieved ≥ 50% reduction, and 13% had worsened. No correlation between change in imaging and change in pain scores
Zhu[18], 2020	n = 200, knee OA with effusion synovitis	RCT (12 months)	5 mg of MTX increases to 15 mg per week, or placebo	Protocol, no results
Kvien[19], 2023	n = 170, erosive hand OA with synovitis	RCT (12 months)	15 mg of MTX increased to 20 mg per week, or placebo	Protocol, no results
HCQ				
Lee[20], 2018	n = 196, symptomatic hand OA	RCT (24 weeks)	400 mg of HCQ per day, or placebo	HCQ had no effect on symptoms of hand OA
Kedor[21], 2021	n = 153, inflammatory and erosive hand OA	RCT (12 months)	200–400 mg of HCQ per day, or placebo	HCQ was not effective for changes in pain, function, and radiographic scores
Saviola[22], 2012	n = 38, erosive hand OA	RCT (12 months)	200–400 mg of HCQ per day or placebo	HCQ had no effect on active erosive hand OA
Kingsbury[23], 2018	n = 248, hand OA with 94% having synovitis	RCT (12 months)	200–400 mg of HCQ per day, or placebo	HCQ was not effective for pain relief. Baseline structural damage or synovitis did not affect the treatment response
Bonfante[24], 2008	n = 29, knee OA	RCT (4 months)	400 mg of HCQ per day for 4 months or placebo	HCQ was not effective for pain relief
Jokar[25], 2013	n = 44, mild to moderate knee OA	RCT (6 months)	200 mg of HCQ twice per day for 6 months or placebo	HCQ improved symptoms in mild to moderate knee OA
Abou-Raya[26], 2014	n = 166, knee OA with clinical signs of synovitis	RCT (9 months)	400 mg of HCQ per day or placebo	HCQ improved pain, function, and synovitis

HCQ, hydroxychloroquine; MTX, methotrexate; OA, osteoarthritis; RCT, randomized controlled trial; NRS, numerical rating scale; WOMAC, Western Ontario and McMaster Universities Osteoarthritis Index

**Table 2:** Biologic agents for the treatment of osteoarthritis

Studies	Participants	Design	Intervention	Conclusion
Tumor necrosis factor-α (TNF-α) inhibitors				
Fioravanti[27], 2009	n = 10, erosive hand OA	RCT (12 months)	0.2 mg of infliximab monthly or saline (placebo)	Infliximab improved both joint symptoms and disease progression after 1 year
Chevalier[28], 2015	n = 85, hand OA	RCT (6 months)	40 mg of Adalimumab for two subcutaneous at a 15-day interval, or placebo	Adalimumab was not superior to placebo to alleviate pain in patients with hand OA not responding to analgesics and NSAIDs
Aitken[29], 2018	n = 43, erosive hand OA with synovitis	RCT (8 months)	40 mg of adalimumab injections every other week or placebo, after an 8-week washout then crossed over treatment groups for 12 weeks or placebo	Adalimumab did not show any effect on pain, synovitis or bone marrow lesions (BMLs)
Kloppenburg[30], 2018	n = 90, erosive hand OA	RCT (12 months)	50 mg of Etanercept per week reduced to 25 mg per week or placebo	Etanercept did not relieve pain effectively in erosive hand OA, but small subgroup analyses showed a signal for effects on subchondral bone in actively inflamed joints
Verbruggen[31], 2012	n = 60, erosive hand OA	RCT (40 months)	40 mg of adalimumab subcutaneously every two weeks during a 12-month or placebo	Adalimumab showed no effect on pain, synovitis, or BMLs in patients with erosive hand OA
Wang[32], 2018	n = 56, moderate to severe knee OA	Open-label RCT (1 months)	10 mg of Adalimumab or 25 mg hyaluronic acid, both received 200 mg of celecoxib per day	Adalimumab had greater pain relief effects compared with hyaluronic acid
Interleukin-1 inhibitors				
Cohen[33], 2011	n = 68 (part A) or n = 160 (part B), knee OA	RCT (6 months)	75 or 300 mg of AMG 108 subcutaneously or 100 or 300 mg intravenously once every 4 weeks or placebo (part A). 300 mg of AMG 108 subcutaneously once every 4 weeks or placebo (part B)	AMG 108 showed statistically insignificant but numerically greater improvements in pain
Chevalier[34], 2009	n = 170, knee OA	RCT (3 months)	50 or 150 mg of anakinra intraarticular injection, or placebo	Anakinra showed no effect on knee symptoms compared with placebo
Fleischmann[35], 2019	n = 350, knee OA with synovitis	RCT (13 months)	25, 100, or 200 mg of lutikizumab subcutaneously every 2 weeks for 50 weeks or placebo	Lutikizumab limited improvement in the WOMAC pain scores, but not synovitis
Wang[36], 2017	n = 36, knee OA	RCT	0.3, 1, or 3 mg/kg of lutikizumab or placebo every other week in three cohorts, and one cohort received ABT-981(3 mg/kg) or placebo every 4 weeks	Lutikizumab may provide clinical benefit to patients with inflammation-driven OA by reducing the levels of inflammatory markers
Cai[37], 2022	n = 260, knee OA with synovitis	RCT (24 weeks)	50 mg of diacerein twice daily for 24 weeks or placebo	Protocol, no results
Schieker[38], 2020	n = 10,061, 15.6% with OA	Post-hoc analyses from RCT	50 or 100 mg of canakinumab subcutaneously once every 3 months or placebo	Canakinumab reduced the risk of total knee and hip replacement over 3.7 years
Kloppenburg[39], 2019	n = 132, ≥ 1 erosive and ≥ 3 tender and/ or swollen hand joints	RCT (24 weeks)	200 mg of lutikizumab subcutaneously every 2 weeks for 24 weeks or placebo	Lutikizumab showed no effect on pain relief or any imaging outcomes, but seeing a decrease in serum high-sensitivity C reactive protein levels, and IL-1α and IL-β levels

NSAIDs, Nonsteroidal Anti-inflammatory Drugs; TNF, tumor necrosis factor; BMLs, bone marrow lesions; WOMAC, Western Ontario and McMaster Universities Osteoarthritis Index; IL-1, Interleukin-1

**Table 3:** Other treatments for the treatment of osteoarthritis

Studies	Participants	Design	Intervention	Conclusion
Nonsteroidal Anti-inflammatory Drugs (NSAIDs)				
Gallelli[40], 2013	n = 90, patients of total knee arthroplasty	Open-label study (2 weeks)	Diclofenac slow release 75 mg/once day or 75 mg/bid, ibuprofen 600 mg/bid or 600 mg/tid, or celecoxib 200 mg/once day or 200 mg/bid	NSAIDs can improves the quality of life and reduced pro-inflammatory synovial fluid cytokine levels in knee OA patients
Gineyts[41], 2004	n = 201, knee OA	RCT (6 weeks)	2400 mg of ibuprofen per day or placebo	High dose of ibuprofen can partly prevent cartilage and synovial tissue degradation
Xu[42], 2020	n = 99, erosive hand OA	Non-random intervention study (2 weeks)	They were divided into a NSADIs withdrawal group and a control group based on whether they took NSAIDs at baseline	No significant changes in inflammatory US features were seen in patients with erosive hand OA after withdrawal of NSAIDs for 2 weeks
Colchicine				
Davis[43], 2020	n = 59, hand OA	RCT (12 weeks)	0.5 mg of colchicine twice per day or placebo	Colchicine was not effective in pain relief, tender and swollen joint count or grip strength, synovitis grade and CRP compared to placebo
Dossing[44], 2023	n = 100, symptomatic hand OA	RCT (12 weeks)	0.5 mg of colchicine oral twice per day or placebo	Colchicine was not effectively in pain relief compared to placebo, and associated with more adverse events
Das[45], 2002	n = 36, knee OA	RCT (5 months)	0.5 mg of colchicine or placebo added to nimesulide	Colchicine improved VAS knee pain and WOMAC scores
Das[46], 2002	n = 39, knee OA	RCT (5 months)	0.5 mg of colchicine or placebo added to intraarticular steroid	Colchicine improved VAS knee pain and a modified WOMAC index score
Aran[47], 2011	n = 61, knee OA without joint involvement and evidences of chondrocalcinosis	RCT (3 months)	0.5 mg of colchicine twice per day or placebo	Colchicine was more effective on VAS pain compared to placebo
Leung[48], 2018	n = 109, symptomatic knee OA	RCT (16 weeks)	0.5 mg of colchicine twice per day or placebo	Colchicine was not effective on pain relief compared with placebo, although it decreased inflammation and high bone turnover biomarkers
Corticosteroids				
Kvien[49], 2008	n = 83, hand OA	RCT (6 weeks)	1 or 2 mg of prednisolone plus 200 mg of dipyridamole or placebo	Combine prednisolone with dipyridamole reduced AUSCAN pain score in hand OA patients
Wenham[50], 2012	n = 70, hand OA	RCT (4 weeks)	5 mg of prednisolone per day or placebo	Prednisolone had no effect on hand OA pain relief
Kroon[51], 2019	n = 92, hand OA with synovitis	RCT (6 weeks)	10 mg of prednisolone per day or placebo	Prednisolone reduced VAS pain compared with placebo
Keen[52], 2010	n = 36, hand OA	Open-label study (12 weeks)	120 mg of intra-muscular methyl prednisolone	Methyl prednisolone improved VAS pain and clinical symptoms
Fish oil and krill oil				
Kuszewski[53], 2020	n = 152, sedentary overweight/obese older adults	RCT (4 months)	Fish oil (2000 mg DHA + 400 mg EPA/day), curcumin (160 mg/day), or a combination of both or placebo	Fish oil but not curcumin (in combination with fish oil or not) alleviated OA-specific pain and burden
Hill[54], 2016	n = 202, knee OA	RCT (24 months)	15 mg of high dose (4.5 g omega-3 fatty acids) or low dose (blend of fish oil and sunola oil; ratio of 1:9, 0.45 g omega-3 fatty acids) per day	Both of them was effective in WOMAC pain, but low dose group had greater improvement in WOMAC pain compared with high dose group, there was no difference between the groups in cartilage volume loss over 2 years
Deutsch[55], 2007	n = 90, cardiovascular disease and/or RA and/or OA and with increased CRP levels	RCT (1 months)	300 mg of NKO™ or placebo per day or placebo	NKO™ significantly inhibited inflammation and reduced arthritic symptoms within a short treatment period
Suzuki[56], 2016	n = 50, mild knee pain	RCT (1 months)	2 g of krill oil per day or placebo for 30 days or placebo	Krill oil improved the subjective symptoms of knee pain in adults with mild knee pain
Stonehouse[57], 2022	n = 235, mild to moderate knee OA	RCT (6 months)	4 g of krill oil (0.60 g EPA, 0.28 g DHA, 0.45 mg astaxanthin) per day or placebo	Krill oil modestly improved knee pain, stiffness, and physical function in adults with mild-to-moderate knee OA
Laslett[58], 2024	n = 260, knee OA, significant knee pain, and effusion synovitis	RCT (6 months)	2 g of krill oil per day or placebo for 6 months	Krill oil had no effect in knee OA with synovitis compared with placebo
CL				
Srivastava[59], 2016	n = 160, keen OA	RCT (4 months)	500 mg of CL extract or placebo twice per day	Chronic use of CL can inhibit inflammatory response, improve clinical symptoms of KOA patients, and reduce oxidative stress
Wang[60], 2020	n = 70, knee OA with effusion–synovitis	RCT (3 months)	2 capsules (2*500 mg) of CL or placebo per day for 12 weeks	CL was more effective in knee pain but not in effusion-synovitis compared with placebo
CS and GS				
Zegels[61], 2013	n = 353, knee OA	RCT (3 months)	1200 mg once daily and 400 mg three daily of CS or placebo	CS was more effective in OA clinical symptoms relief measured by Lequesne index compared to placebo
Wildi[62], 2011	n = 69, knee OA with synovitis	RCT, Open-label (12 months)	800 mg of CS or placebo per day for 6 months, then 800 mg of CS in both 2 groups for 6 months of the open-label phase	CS treatment significantly reduced the cartilage volume loss in knee OA starting at 6 months of treatment, and bone marrow lesions at 12 months
Rozendaal[63], 2008	n = 222, hip OA	RCT (2 years)	1500 mg oral daily of GS for 2 years or placebo	GS was no more effective in reducing symptoms and progression of hip OA
McAlindon[64], 2004	n = 205, knee OA	RCT (12 weeks)	1.5 g per day of glucosamine or placebo	Glucosamine was no more effective in treating the symptoms of knee OA compared to placebo
Giordano[65], 2009	n = 60, knee OA	RCT (12 weeks)	1500 mg per day of GS or placebo	GS was more effective in pain and function improvements compared to placebo
Roman-Blas[66], 2017	n = 164, knee OA	RCT (6 months)	1200 mg CS plus 1500 mg GS per day or placebo for 6 months	CS plus GS was no more effective in pain and function relief in knee OA at 6 months
Clegg[67],2006	n = 1583, symptomatic	RCT (24 weeks)	500 mg GS, 400 mg CS, 500 mg GS plus 400 mg CS, 200 mg celecoxib or placebo three times per day	GS, CS or their combine was no more effective in pain relief compare to placebo
Fransen[68], 2015	n = 605, knee pain and medial tibiofemoral compartment narrowing	RCT (24 months)	400 mg of CS plus 753 mg of GS per day or placebo	CS plus GS significantly reduced the JSN but not pain

DHA, docosahexaenoic acid; EPA, eicosapentaenoic acid; RA, rheumatoid arthritis; CRP, C-reactive protein; CL, Curcuma longa; CS, Chondroitin sulphate; GS, glucosamine sulphate; JSN, joint space narrowing; NSAIDs, Nonsteroidal Anti-inflammatory Drugs

## Disease-modifying antirheumatic drugs (DMARDs)

### Methotrexate

Methotrexate (MTX) is one of the first-line disease-modifying antirheumatic drugs (DMARDs) commonly used to treat synovitis in inflammatory arthritis, especially rheumatoid arthritis (RA), with satisfactory effectiveness and long-term safety [69]. The mechanisms of action of the anti-synovitis effect of methotrexate have been well documented [70]. It is hypothesized that MTX could also reduce synovitis and pain in inflammatory OA. Erosive hand OA is considered a form of inflammatory OA with poorer clinical and radiographic outcomes than non-erosive hand OA [71]. In a small open-label, single-arm study of 21 patients with erosive hand OA (published as a conference abstract only), the authors found that a weekly dose of 10 mg MTX decreased pain, stiffness, and functional disability, although number of tender and swollen joints did not change[11]. Similar findings were observed in a randomized controlled trial (RCT) of 64 patients with erosive hand OA showing that the same dose of MTX reduced visual analogue scale (VAS, 0–100, a higher score indicates worse pain) pain within the treatment group [12]. The reduction in pain was greater (albeit not statistically significant) in patients receiving MTX compared to placebo over 3 (-21.1 vs -11.7) but not 12 months (-14.3 vs -17.8) [12]. Neither trial limited their participants to having synovitis, and the later trial reported a very low prevalence of synovitis (29 of 1024 joints), which may have underestimated the effect of MTX [12]. In a recent RCT of 97 patients who had hand OA and MRI-detected synovitis, 20 mg of MTX per week showed a moderate and potentially clinically meaningful effect on relieving VAS pain and the Australian Canadian Osteoarthritis Hand Index (AUSCAN) stiffness but not function over a 6-month period compared to placebo [13]. The effect of MTX on radiographic progression and tender swollen joint count over 2 years was not able to be evaluated due to the COVID-19 pandemic [13].

For knee OA, a 6-month RCT showed that a weekly dose of 15 mg MTX reduced pain severity, functional status, and quality of life over 3 and 6 months in 100 patients with moderate to severe (KL score 3–4) knee OA compared to placebo[14]. However, another 4-month RCT did not observe any differences in change in pain and functional limitation between the 7.5 mg MTX and placebo groups, although there was a tendency to an increased consumption of paracetamol in the placebo group [15]. Preliminary findings from a multicenter RCT indicated that high dose of MTX (maximum 25 mg) improved 0–10 Numeric Rating Scale (NRS) knee pain (adjusted treatment difference -0.83 (95% CI -1.55 to -0.10), Western Ontario and McMaster Universities Osteoarthritis Index (WOMAC) stiffness and function, but not WOMAC pain at 6 months compared to placebo (published in a conference abstract only) [16, 72]. While the effect on pain was moderate, it was below a clinically meaningful threshold, did not persist at 12 months and there were no changes in synovial volume [16]. The participants in this trial had clinical and radiographic knee OA but were not selected based on inflammation[72]. The variation in therapeutic efficacy of MTX across these studies may be attributed to differences in dosage. In an open-label, single-arm trial of 30 patients with symptomatic knee OA and effusion synovitis on ultrasound, 43% of patients achieved ≥ 30% reduction in knee pain after MTX treatments (20 mg weekly) over 24 weeks but there was no correlation between change in knee pain and change in synovitis thickness [17]. Given this was an open-label study with only one treatment group, potential placebo effects may have been overlooked. Although no formal RCTs have evaluated the effect of MTX on the structural progression of OA, current studies suggested that MTX can relieve joint symptoms and that a stronger effect in patients with synovitis is likely but needs to be confirmed. Several ongoing RCTs conducted in OA patients with synovitis will answer these questions [18, 19, 72]. High-dose MTX demonstrates promising analgesic effects in both hand and knee OA, with a good safety profile when used in conjunction with folic acid supplementation. Additionally, it may yield greater efficacy in patients with hand OA accompanied by synovitis, but it is still unclear in knee OA.

### Hydroxychloroquine

Hydroxychloroquine (HCQ), an anti-malarial drug, is widely used in RA and systemic lupus erythematosus [73]. The therapeutic effect of HCQ may be related to its anti-inflammatory activity, including the inhibition of T-cell activation and cytokine release [74]. Increasing evidence suggests that these pathways may also be involved in OA [75], supporting HCQ as a potential treatment for inflammatory OA.

For hand OA, current evidence consistently suggests a lack of efficacy of HCQ on hand pain. In a recent RCT of 196 patients with symptomatic hand OA, pain relief was similar in the HCQ and placebo groups over 24 weeks [20]. Another RCT involving 153 patients with inflammatory (based on clinical symptoms) and erosive hand OA also showed that HCQ was not different from placebo in symptom relief [21]. Consistently, an RCT evaluating the efficacy of HCQ in erosive hand OA similarly demonstrated that 400 mg of HCQ for 30 days, followed by a reduction in dosage to 200 mg for 11 months, was not effective on VAS pain or Dreiser’s score [22]. It is worth noting that patients in the HCQ group reported poorer compliance, which could also influence the study results. In an RCT of 248 patients with hand OA and most of them had synovitis (134 of 143) in at least one joint by ultrasound, HCQ (200 to 400 mg of dosing according to weight) was no better than placebo for pain relief after 12 months [23]. No dose–response relationship was found for the efficacy, and there was no correlation observed between the presence of synovitis detected by power Doppler and the magnitude of the treatment effect.

For knee OA, the findings from two RCTs are inconsistent regarding the effect of HCQ on knee symptoms. In a small RCT of 29 patients with symptomatic and radiographic knee OA, 400 mg of HCQ per day for 4 months did not improve knee symptoms as assessed by WOMAC, Lequesne index, and VAS, compared to placebo [24]. In another RCT of 44 patients with knee pain and radiographic knee OA, however, 200 mg of HCQ twice per day significantly improved WOMAC pain (-12.24 VS -0.91, range 0–50), function (-29.90 VS -1.56, range 0–170), and stiffness (-2.24 VS -0.74, range 0–20) over 24 weeks compared to placebo [25]. In addition, consumption of painkillers was significantly higher in the placebo group than in the HCQ group.

In an RCT of 166 patients with moderate to severe knee OA and synovitis, detected clinically and by ultrasound, 400 mg of HCQ per day for 9 months reduced both VAS knee pain and synovitis compared to the placebo group (published as a conference abstract only) [26]. These studies imply that efficiency of HCQ in knee OA is inconsistent and thus further high-quality evidence is needed. In a recent meta-analysis of 6 RCTs of patients with knee or hand OA, pooled results showed no effect of HCQ on pain and function of hand or knee OA [76].

## Biologic agents

### Tumor necrosis factor-α (TNF-α) inhibitors

TNF-α is a proinflammatory cytokine that can be produced by a variety of cells including synovial cells and chondrocytes in OA [77]. In addition, TNF-α can directly stimulate osteoclast differentiation and enhance the expression of a series of proinflammatory cytokines [78]. Previous studies have shown that TNF-α is associated with OA pain [79]. TNF-α inhibitors, such as infliximab, adalimumab, and etanercept, suppress the immune system and inflammation by blocking the activity of TNF-α [80].

In a small pilot study of 10 women with erosive hand OA, 0.2 mg of infliximab per month improved both joint symptoms and disease progression after 1 year compared to saline (placebo) [27]. However, in an RCT of 85 patients with painful hand OA refractory to analgesics and NSAIDs, two subcutaneous injections of 40 mg adalimumab at a 15-day interval were not effective for pain relief after 6 weeks and 6 months, compared to placebo [28]. While this study did not restrict the participants to have synovitis, another crossover trial of 43 participants with erosive hand OA and MRI-detected synovitis showed similar results, that 40 mg of adalimumab (injections every other week) did not improve hand pain or synovitis after 12 weeks [29]. Moreover, in an RCT of 90 patients with erosive OA, 50 mg per day of etanercept for 24 weeks thereafter 25mg per day did not demonstrate significant pain relief after 1 year compared to placebo [30]. However, in prespecified per-protocol analyses, etanercept improved hand pain in patients who complied with the treatment and had pain and soft swelling, power Doppler signal, or MRI-detected synovitis at baseline [30]. Moreover, etanercept treatment was associated with a higher rate of radiographic remodeling and fewer bone marrow lesions, especially in joints with inflammation at baseline [30]. Consistently, another RCT of 60 patients with erosive hand OA also found that 40 mg of adalimumab every 2 weeks slowed erosive progression after 1 year [31].

For knee OA, except for a case report showing that adalimumab improved knee pain, synovitis, and bone marrow lesions after 6 months [81], there was only one open-label RCT including 56 knee OA patients assessing the efficacy of adalimumab compared with hyaluronic acid in moderate to severe knee OA [32]. Patients who received 10 mg of adalimumab for 4 weeks significantly improved VAS scores, WOMAC scores, Global Assessment (PGA), and Physician Global Assessment (PhGA) compared to those who received 25 mg of hyaluronic acid [32].

The findings from these RCTs suggest that TNF-α inhibitors may exhibit a therapeutic effect on both joint symptom and structural progression of hand OA over the long term (e.g. 1 year), especially in patients with a high level of inflammation. However, this needs to be confirmed in larger and longer RCTs. Low-level evidence suggests that TNF-α inhibitors may be effective for knee OA, but it is unclear whether the effect is stronger in patients with synovitis and there were no rigorously conducted RCTs to date.

### Interleukin-1 inhibitors

Interleukin-1α (IL-1α) and IL-1β are proinflammatory cytokines that have similar effects as TNF-α in inflammatory arthritis, including involvement in structural damage and symptoms [82]. However, the performance of IL-1 inhibitors in RCTs for OA has been unsatisfactory. In an RCT of 228 patients with knee OA, AMG 108, an inhibitor of both IL-1α and IL-1β, did not significantly improve WOMAC pain scores (range 0–500) after 12 weeks compared to placebo (median change, -63 vs -37, p = 0.25) [33].

Another RCT including 170 patients with symptomatic knee OA showed similar results that intra-articular injection of anakinra (an IL-1 receptor antagonist), either 50 or 150 mg, had no effect on knee symptoms compared to placebo [34], and no significant improvement was found in cartilage degradation markers. In vitro studies have shown that at least 10–100 times excessive doses are required to affect the activity of IL-1β in cartilage [83]. It is important to note that neither trial, which evaluated the effect of anti-inflammatory treatments, restricted their participants to those with an OA inflammatory phenotype. Two RCTs sought to overcome this limitation by recruiting participants with OA and synovitis [35, 37]. One of them was a phase 2 trial that investigated the effect of lutikizumab on knee symptoms in 350 patients with radiographic knee OA and synovitis detected on ultrasound or MRI [35]. Lutikizumab is a novel human dual variable domain immunoglobulin that has shown better performance in pain and cartilage damage, compared to inhibition of IL-1α or IL-1β alone in a mouse model of OA [84]. In the phase 2 trial, however, lutikizumab of different doses (25, 100, or 200 mg subcutaneously every 2 weeks) did not show satisfactory effect on pain and synovitis (as measured by synovial membrane thickness, synovial fluid volume, and WORMS synovitis/effusion score) compared with placebo, only the 100 mg group showed a significant improvement in WOMAC pain at 16 weeks compared with placebo (P = 0.050) [35]. However, subsequent analysis revealed that patients with K/L grade 3 knee osteoarthritis continued to experience superior pain relief compared to placebo from 16 to 52 weeks. This suggests that patients with severe knee OA may derive greater benefit from treatment with lutikizumab. Meanwhile, in a phase I trial of patients with knee OA, lutikizumab treatment was associated with a reduction in serum levels of inflammatory biomarkers [36]. However, the decrease in the levels of inflammatory biomarkers did not result in additional therapeutic benefits. The inconsistent results between animal studies and human trials were thought to be due to different disease stages, severity of synovitis, and route of administrating IL-1 inhibitors [85]. A trial is underway to evaluate the effect of diacerein on knee pain and effusion-synovitis in patients with symptomatic knee OA and MRI-detected effusion-synovitis [37]. Diacerein is an oral IL-1β inhibitor which has been shown to be beneficial for pain and joint space narrowing (JSN) in OA patients, according to a systematic review of clinical trials [86]. Interestingly, in a post-hoc analysis of a large RCT (n = 10,061), canakinumab, an IL-1β inhibitor, reduced the risk of total knee and hip replacement surgeries and OA-related adverse events over 3.7 years, in participants with or without OA at baseline [38]. Previous studies have also indicated that IL-1β may play a role in driving cartilage degradation [87]. These findings suggest that IL-1 inhibitors may exhibit a potential role in the structural progression of OA.

Little is known about the effect of IL-1 inhibitors on hand OA. In an RCT of 132 patients with erosive hand OA and moderate to severe inflammation, confirmed by tender and/or swollen interphalangeal and synovitis by MRI at baseline, lutikizumab (200 mg every two weeks) did not improve the AUSCAN pain score or any imaging outcomes (e.g. JSN, osteophytes, synovitis, BMLs) after 26 weeks [39]. This is despite seeing a decrease in serum high-sensitivity C reactive protein levels, and IL-1α and IL-β levels [39], suggesting that even though lutikizumab affects inflammatory biomarkers this is not resulting in pain relief for patients.

The lack of desired pain relief with IL-1 inhibitors may be attributed to the possibility that, although synovium is a significant source of IL-1, IL-1 itself may not be essential for sustaining synovial inflammation. Exploring and discovering other factors such as cartilage degradation products and adipokines[9] associated with synovial inflammation may yield greater benefits.

## Other treatments

### Nonsteroidal Anti-inflammatory Drugs (NSAIDs)

The effect of both oral and topical Nonsteroidal Anti-inflammatory Drugs (NSAIDs) on symptomatic OA has been well documented in RCTs, although there are differences in effectiveness and safety among various NSAIDs [88]. In clinical practice, NSAIDs are widely used in OA patients to relieve joint symptoms, and they are recommended by the most up-to-date guidelines for the treatment of OA [89, 90].

NSAIDs alleviate pain and inflammation by inhibiting the synthesis of prostaglandins [91]. Both animal studies and in vitro experiments show a potential chondroprotective effect of NSAIDs [92, 93]. However, there is a scarce amount of RCTs to evaluate whether the effect of NSAIDs is more pronounced in patients with inflammatory OA. In an open-label trial of 90 patients scheduled for a total knee arthroplasty, high dosages of 3 common NSAIDs (i.e. celecoxib, diclofenac, and ibuprofen), compared to low dosages, significantly improved WOMAC score and decreased the concentration of inflammatory cytokines in the synovial fluid 14 days after beginning of treatment, suggesting a short-term effect of NSAIDs on both OA symptoms and inflammation [40]. In a placebo-controlled trial of 201 patients with knee pain, radiographic OA, and joint inflammation (documented by knee swelling), 2400 mg/day of ibuprofen prevented the increase of markers reflecting cartilage and synovium metabolism after 4 to 6 weeks of treatment, compared to placebo [41]. However, the effect on inflammatory markers does not necessarily translate to improvements in joint structures. Using data from 990 participants with moderate to severe knee OA in the Osteoarthritis Initiative, Luitjens et al. found no beneficial effect of NSAIDs on change in synovitis or cartilage thickness or composition over 4 years [94]. Moreover, in another study of 99 patients with erosive hand OA, cessation of NSAIDs over 2 weeks did not significantly change inflammatory features (i.e. synovial proliferation, effusion and power Doppler signal) on ultrasound compared with patients who did not regularly take NSAIDs [42]. As one of the most used anti-inflammatory treatments for OA, NSAIDs have well-known adverse effects affecting the gastric mucosa, renal system, cardiovascular system, hepatic system, and hematologic system [95]. Further studies are warranted to understand whether the effect of NSAIDs differs among patients with inflammatory OA and those without, thereby optimizing the use of NSAIDs in OA treatment.

### Colchicine

Colchicine is an anti-inflammation drug often used to treat gout, which has the potential to prevent uric acid crystal-induced inflammation that exists in OA [96].

For hand OA, an RCT of 59 patients aged 40–80 years showed that 0.5 mg of colchicine twice per day was not effective on pain relief, tender and swollen joint count, or grip strength and synovitis grade compared to placebo for 12 weeks [43]. Similar results were observed in a recent RCT of 100 symptomatic hand OA patients, in which 0.5 mg of colchicine twice per day was not more effective on pain relief compared to placebo for 12 weeks, and the treatment led to more adverse events [44].

For knee OA, two pilot RCTs from the same group published in 2002 (n = 36 and 39, respectively) showed that colchicine added to nimesulide or intraarticular steroid improved VAS knee pain and WOMAC symptoms over 20 weeks compared to nimesulide or intraarticular steroid alone [45, 46]. These encouraging findings were repeated by one follow-up trial but not another.

An RCT of 61 postmenopausal patients with primary knee OA suggested that 0.5 mg of colchicine twice per day for 3 months can significant improved VAS pain score compared to placebo [47]. Another RCT of 109 symptomatic knee OA patients indicated that 0.5 mg of colchicine twice per day for 16 weeks was not effective on WOMAC scores although the mean levels of serum high-sensitivity C-reactive protein (hs-CRP) and synovial fluid C-terminal crosslinked telopeptide of Type I collagen were decreased compared with placebo [48]. The study quality of available evidence was low overall, and no RCT has specified the patients to have a sign of inflammation, for whom the anti-inflammatory property of colchicine may bring greater benefits.

### Corticosteroids

Intra-articular corticosteroids are often used to improve the symptoms in OA patients [97]. However, the role of corticosteroids remains unclear in OA. One possible explanation is that corticosteroids may achieve analgesic purposes by inhibiting synovial inflammation [98]. In a trial evaluating the effect of a combination of prednisolone and dipyridamole, CRx-102 (comprises dipyridamole and low dose prednisolone) reduced AUSCAN pain scores in 83 hand OA patients[49]. Meanwhile, a previous study has shown that 120 mg of methylprednisolone intramuscular injection was effective on inflammatory hand pain [99]. At baseline, 55% of patients had ultrasound-detected synovitis and 23% had clinical synovitis. Interestingly, further analysis showed that ultrasound-detected synovitis was associated with treatment response, but not clinical synovitis. This seems to imply that methylprednisolone is more effective in patients with early synovitis.In an RCT of 70 participants with hand OA and 75% had MRI-detected synovitis, 5 mg of oral prednisolone per day was not effective in relieving VAS pain compared with placebo for 4 weeks [50]. The lack of analgesic effect may be due to the low dose. Another RCT included 92 patients with symptomatic hand OA and ultrasound-detected synovitis, and 10 mg of oral prednisolone daily significantly improved VAS hand pain compared to placebo (-21.5 vs -5.2). Moreover, synovitis thickening (by ultrasound) and BMLs but not soft tissue swelling or synovitis on MRI were also improved in the prednisolone group [51]. An Open-label study of 36 hand OA patients showed that 120 mg of intra-muscular methylprednisolone improved VAS pain and clinical symptoms but not grey-scale synovitis over 12 months [52]. This may be due to the fact that in the participants of this study, gray-scale synovitis on ultrasound was usually of low grade, and short-term treatment effects may be easily overlooked. Further analysis showed that patients who met the criteria for an Osteoarthritis Research Society International response criteria had more gray-scale synovitis and power Doppler signals at baseline. These findings suggest that corticosteroids may have a greater effect in inflammatory hand OA.

For knee OA, the role of corticosteroids in knee OA with synovitis is unclear. There was an RCT showing that intra-articular triamcinolone did not reduce knee pain and even resulted in more cartilage loss over 2 years in patients with symptomatic knee OA [100].

### Fish oil and Krill oil

Fish oil and krill oil are rich in omega-3 fatty acids, such as eicosapentaenoic acid (EPA) and docosahexaenoic acid (DHA) [101]. Omega-3 fatty acids can compete with omega-6 fatty acids to reduce pain, swelling, neutrophil, and metalloproteinase activation, thereby alleviating synovitis and delaying the progression of cartilage degeneration [102]. There is evidence that these supplements may be beneficial for synovitis and cartilage damage [54, 101].

Fish oil has been widely used in the combined treatment of RA. A literature review suggested that fish oil can relieve pain and morning stiffness and reduce the intake of non-steroid anti-inflammatory drugs in patients with RA [103]. Fish oil has also been used for OA with some efficacy [54, 104]. In an RCT of 152 overweight/obese older adults with OA pain (site not specified) measured by the short-form McGill Pain Questionnaire, fish oil reduced OA-specific pain and burden of OA over 16 weeks [53]. A separate study which evaluated high-dose fish oil compared to low-dose fish oil found that pain improved in both groups but that the low-dose fish oil group demonstrated the greatest improvement in WOMAC pain and function scores at 2 years [54]. There was no difference between the groups in cartilage volume loss or CRP levels over 2 years [54]. There were no studies to evaluate the effect of fish oil in OA patients with synovitis. Further studies are needed to confirm the role of fish oil in OA patients with synovitis.

Krill oil is also rich in EPA and DHA, and it contains antioxidants such as astaxanthin [101]. Krill oil had better effects than fish oil in experimental RA models [105]. There were 2 small RCTs (90 and 50 participants, respectively) indicating that krill oil can relieve pain, function, and inflammation in patients with knee OA [55, 56]. Another RCT involving 235 patients with OA showed that 4 g daily of krill oil (0.60 g EPA, 0.28 g DHA and 0.45 mg astaxanthin) provided modest pain relief over 6 months in patients with moderate to severe knee OA [57]. While these studies suggested that krill oil may be moderately effective on knee symptoms in general OA patients, a recent RCT of 262 patients with knee OA and synovitis showed no effect of 2 g of krill oil (0.19 g EPA and 0.1 g DHA) on pain or synovitis over 24 weeks [58]. Although the difference in efficacy between the two studies could be attributed to the dose, neither study reported any improvement in local inflammation. This suggests that the analgesic effect of krill oil, if any, may not be through its anti-inflammatory effect.

### *Curcuma longa* extract

Curcuma longa extract (CL) is frequently used in traditional Chinese medicine and Ayurveda for the treatment of OA through its anti-inflammatory, antioxidant, and analgesic properties [106]. In an RCT of 160 patients with knee OA, 500 mg of CL extract per day suppressed biomarkers of both inflammation and oxidative stress and reduced knee pain compared to placebo [59]. The lack of improvement in radiographic manifestations of the joints after four months of treatment suggests that the alleviation of pain with CL may be attributed to reduced inflammation. This implies that CL extract may be more effective in patients with an inflammatory phenotype of OA. A systematic review of RCTs has shown that CL extract is beneficial for pain relief in patients with knee OA [107], although these trials did not include patients with inflammatory OA. In a recent RCT of 70 patients with symptomatic knee OA and ultrasound-defined effusion-synovitis, 2 capsules (2*500 mg) of CL extract significantly improved knee pain after 16 weeks [60]. However, unexpectedly, pain relief was only observed in patients with smaller but not larger effusion-synovitis at baseline. This contrasts with the hypothesis that CL extract may be more effective in patients with an inflammatory phenotype of OA. This may be due to that those with a higher effusion-synovitis volume had more severe disease, for which CL extract was less effective. Despite inconsistent dosages and slight variations in study design (regarding co-administration with nonsteroidal anti-inflammatory drugs), the current findings suggest that CL is moderately effective and safe for knee osteoarthritis. However, its potential to target synovitis for OA treatment remains ambiguous.

### Chondroitin sulfate and glucosamine sulfate

Chondroitin sulfate (CS) and glucosamine sulfate (GS) are a glycosaminoglycans that has long been found to have anti-inflammatory and cartilage-protective effects in OA [108]. Animal and clinical studies suggested that CS can inhibit the progression of synovitis [109, 110]. A previous meta-analysis of RCTs has indicated that CS may be superior to placebo in pain relief for hip, knee and hand OA patients [111]. In an RCT of 353 patients with knee OA showed that both 1200 mg once daily and 400 mg three daily of CS was more effective in OA clinical symptoms relief measured by Lequesne index compared to placebo [61]. Another RCT of 69 patients with knee OA and clinical signs of synovitis (warmth, swelling, or effusion), CS 800 mg for 6 months significantly reduced cartilage volume loss at 6 months and bone marrow lesions at 12 months, compared to placebo [62].

The effect of GS in OA is controversial. Several RCTs showed that GS may be no effect in pain relief for hip and knee OA [63, 64]. In contrast, another RCT of 205 patients with knee OA showed that 1500 mg of GS daily was more effective in WOMAC pain, stiffness and physical function score for knee OA compared to placebo [65]. Compared to this study, the two negative studies did not exclude other rheumatic diseases, which could potentially influence the source of joint pain [63, 64]. Furthermore, one study suggests that variations in the formulation of GS may contribute to differences in efficacy [112], which could partially account for discrepancies in therapeutic outcomes. In addition, CS is often used in combination with GS [113]. But the results are controversial similarly. In an RCT of 164 patients with knee OA and moderate to severe pain, CS 1200 mg plus GS 1500 mg daily did not reduce VAS pain over 6 months [66]. Similarly, another large RCT that used the same dose showed that neither CS nor GS or their combination reached a greater proportion of pain relief by 20% compared to placebo over 24 weeks in 1583 patients with moderate-to-severe knee pain [67]. This is despite that another RCT involving 605 patients with knee OA showed that the CS 400 mg plus GS 753 mg significantly reduced JSN but not pain over 24 months [68]. As patients with synovitis tend to have higher levels of pain, a combination of CS and GS may achieve better outcomes in patients with synovitis. Interestingly, although studies of CS and GS have been mostly positive, neither of these treatments is recommended for the treatment of OA due to potential publication bias[114].

## Conclusion

Inflammatory OA is a common and important phenotype of OA that may benefit more from anti-inflammatory treatments but to date there are limited high-quality RCTs which have selected OA patients based on inflammation. Current evidence suggests that MTX, TNF-α inhibitors and prednisolone may have a better efficacy on pain relief in patients with hand OA and synovitis. However, it is uncertain whether anti-inflammatory treatments have a stronger effect in pain relief for patients with knee OA and an inflammatory phenotype.

### Supplementary Information

Below is the link to the electronic supplementary material.

Supplementary file1 (XLSX 36 KB)

## Data Availability

Not applicable.
